# Assessing the validity of test scores using response process data from an eye-tracking study: a new approach

**DOI:** 10.1007/s10459-022-10107-9

**Published:** 2022-05-05

**Authors:** Victoria Yaneva, Brian E. Clauser, Amy Morales, Miguel Paniagua

**Affiliations:** National Board of Medical Examiners, 3750 Market Street, Philadelphia, PA 19104-3102 USA

**Keywords:** Eye tracking, Machine learning, Score interpretation, Validity

## Abstract

Understanding the response process used by test takers when responding to multiple-choice questions (MCQs) is particularly important in evaluating the validity of score interpretations. Previous authors have recommended eye-tracking technology as a useful approach for collecting data on the processes test taker’s use to respond to test questions. This study proposes a new method for evaluating alternative score interpretations by using eye-tracking data and machine learning. We collect eye-tracking data from 26 students responding to clinical MCQs. Analysis is performed by providing 119 eye-tracking features as input for a machine-learning model aiming to classify correct and incorrect responses. The predictive power of various combinations of features within the model is evaluated to understand how different feature interactions contribute to the predictions. The emerging eye-movement patterns indicate that incorrect responses are associated with working from the options to the stem. By contrast, correct responses are associated with working from the stem to the options, spending more time on reading the problem carefully, and a more decisive selection of a response option. The results suggest that the behaviours associated with correct responses are aligned with the real-world model used for score interpretation, while those associated with incorrect responses are not. To the best of our knowledge, this is the first study to perform data-driven, machine-learning experiments with eye-tracking data for the purpose of evaluating score interpretation validity.

 As computer administration of tests has become widespread, more attention has been given to collateral information collected during administration, often referred to as *process data*. Process data provides information about how the test takers respond that goes beyond whether the response was correct or incorrect. These types of data can provide important evidence to evaluate the validity of intended score interpretations and uses.

Kane and Mislevy (2017) provide a detailed discussion of the role of process data in evaluating validity claims for score interpretations. The basis of the argument is that there should be an alignment between the cognitive process used for problem solving in the real-world setting of interest and the cognitive process used by test takers in responding to test items. While the two processes do not need to be identical, it is important that key features of the process used for real-world responses are necessary for successfully completing the examination task.

Although Kane and Mislevy (2017) argue for the importance of an evaluation of process data as part of an overall validity argument, they note that process data is much more likely to provide a definitive rejection of the intended interpretation of test scores than definitive support. In this context, they repeat a frequently quoted comment from Lee Cronbach, “The job of validation is not to support an interpretation, but to find out what might be wrong with it. A proposition deserves some degree of trust only when it has survived serious attempts to falsify it” (p. 103, Cronbach, 1980). For example, process data showing that test takers run out of time and answer at random near the end of a math test might lead to the rejection of the hypothesis that the resulting test scores can be interpreted as valid measure of math proficiency. If in the same context process data showed that test takers typically had more time than they needed, we might reject the alternative interpretation that the test is speeded, but sufficient time is not evidence in itself that the test appropriately measures the construct of interest. Kane and Mislevy maintain that process data is nonetheless important because a complete validity argument requires both positive evidence supporting the intended interpretation as well as evaluation of alternative interpretations.

In the case of MCQs, understanding the response process used by test takers can be particularly important in evaluating the validity of score interpretations. Consider an example from a math test. An item might instruct the test taker to examine an equation, solve for *x,* and select the corresponding answer from a list of five options. One process that might be used to respond to the question is to solve for *x.* An alternative process is to consecutively substitute each of the five options into the equation until the test taker finds the one for which the equity holds. The first process supports a conclusion about the test taker’s algebra skills; the second probably does not. Evidence of the second process would likely argue against interpreting the scores as a reflection of the test takers proficiency in algebra. Again, evidence that falsifies an alternative interpretation of the scores may be critical to the overall validity argument, even if it does not, in itself, prove that the intended interpretation can be accepted.

One way to provide such evidence is to collect fine-grained process data by tracking the test taker’s eye movements. Eye-tracking data provides a continuous record of an individual’s gaze (i.e., where the individual is looking). Several authors have specifically pointed to the importance of this technology for evaluating the validity of score interpretations (e.g., Gorin, 2006; Oranje et al., 2017) because it provides evidence about the cognitive process that test takers use in responding to test questions (although that evidence may be indirect). In the words of Fitts et al. (1950) who used eye tracking to evaluate cockpit design, “If we know where a pilot is looking, we do not necessarily know what he is thinking, but we know something of what he is thinking about.”

In this paper we investigate whether data-driven approaches to analyzing eye-tracking data can provide a basis for rejection of the intended interpretation of test scores based on clinical MCQs. To provide context for the study, the next section discusses previous work on the use of eye tracking in assessment.

## Previous eye-tracking research in assessment

As we noted, several authors have advocated for using process data from eye tracking as a means of understanding how test takers respond to test questions. In early studies, this simply meant using this technology to distinguish between the time spent reading the item for the first time from the time spent re-reading it and looking for specific information (Hegarty et al., 1992). Later studies report differences in eye movements between more and less proficient individuals in responding to science-based MCQs and suggest that these may be associated with an individual’s level of expertise with the specific topic area (Tai et al., 2006). This was subsequently supported by evidence that students “paid more attention to chosen options than rejected alternatives, and spent more time inspecting relevant factors than irrelevant ones”, with unsuccessful problem solvers experiencing difficulties in decoding the problem and recognizing the relevant factors (Tsai et al., 2011). Hu et al. (2017) also report that low-performing participants adopt a trial-and-error strategy, while high-performing students have fewer fixations across tasks. Finally, Langenfeld et al. (2020) report that unsuccessful problem solvers miss key pieces of information and focus on irrelevant information.

One commonality of these studies is that they all report evidence of differences between high- and low-performing test takers, with many of them deliberately recruiting participants of differing levels of proficiency. Differentiating between the response process used by successful and unsuccessful test takers is important because validity arguments—and challenges to those arguments—are typically based on the idea that the cognitive processes used for a correct response reflect important aspects of the cognitive processes used to respond in the real-world setting. The processes used by unsuccessful test takers are typically less important than those of successful test takers.

Most previous studies employ a small number of eye-tracking features^1^ (usually between 1 and 4), mainly related to fixation durations and their locations. The number of participants is modest in earlier studies (e.g., 6) and somewhat larger in later studies (e.g., 28, 14, 18). The sampling rate of the devices used in these previous studies is 60 Hz (with one exception being 120 Hz in Hu et al. (2017)), meaning that they capture 60 pictures of the eye positions per second.^2^ (See Appendix A for a summary comparison between the studies in terms of number of participants, type of items, and gaze features). In addition, most previous work uses a similar approach in which a hypothesized cognitive model used to develop the items is evaluated by comparing those eye-tracking features that the researchers assume would be relevant in discriminating between more and less proficient test takers. Although this approach has provided useful results, it has significant drawbacks. First, these studies use a small number of eye-tracking features. This may have limited the researchers’ ability to identify fine-grained differences between processes. The second drawback is that the methodology requires an initial hypothesis regarding where the differences between successful and unsuccessful problem solvers may be found, which may miss important information contained in the data. The third drawback is that the exploration of between-participant differences is done at the level of single variables, ignoring the possibility that the interactions between variables may reveal important patterns. In other words, the existing assessment-related eye-tracking studies have generally focused on identifying simple patterns through assumption-based comparisons as opposed to complex interactions using data-driven approaches. Finally, these studies assume that the differences in response process are a characteristic of the test taker, with more proficient test takers using one process and less proficient test takers using another. This ignores the possibility that the same test taker may use different response processes for different items.

## The proposed approach

The present study addresses the potential drawbacks of previous work by presenting an eye-tracking study to evaluate the problem-solving strategies of successful and unsuccessful test takers. This work utilizes a data-driven machine-learning approach to extract response patterns from a large number of gaze-based features. Identification of the process features that discriminate between correct and incorrect responses is not determined by a working hypothesis but by training an algorithm to use the gaze data as input to predict whether the selected response is correct or incorrect. This approach allows us to determine: (1) whether the eye-tracking features contain signal useful for predicting whether the response is correct, and (2) what feature combination is most predictive of the outcome variable. In this context, the purpose of the analysis is not to make the prediction but to identify the response processes that are associated with correct and incorrect responding. Again, we are not looking for differences associated with varying levels of proficiency; instead, we are looking for patterns associated answering correctly (admittedly the two phenomena are related).

The stimuli used in the study are MCQs designed to assess clinical reasoning in physicians in training. Each test item presents information about a patient and asks the test taker to identify the most likely diagnosis or make other relevant decisions based on that information. The professionally developed test items we studied have been written to reflect content and a cognitive challenge that is relevant to real-world clinical reasoning. As we have noted, eye tracking cannot tell us *what* the test taker is thinking, but it may tell us that the process used to respond is inconsistent with our expectations for real-world clinical reasoning. Evidence to support a potential alternative interpretation that correct responses to the test items use a cognitive process that is inconsistent with real-world clinical reasoning would seriously undermine the validity argument. If this alternative interpretation is not supported by evidence, its credibility is reduced; this in turn means that the credibility of the intended interpretation is at least implicitly supported. Of course, to use this process data to evaluate the validity of score interpretations requires that we begin with a cognitive model for the real-world behavior of interest. Without this model we have no basis for evaluating the process used in responding to the test questions. We describe that model in the next section.

## A cognitive model for clinical reasoning

Kane and Mislevy distinguish between *strong* and *weak* cognitive models of the process used for problem solving. In general, strong cognitive models are only available for highly circumscribed tasks such as simple arithmetic (Tatsuoka, 1983) or other problems for which definitive rules can be established (Carpenter et al., 1990). For more complex and ill-defined activities such as clinical reasoning, weaker, more general models must suffice.

In the absence of a strong cognitive model, we rely on the simpler conceptual framework for clinical diagnostic reasoning presented by Bowen (2006) for this study. This model begins with data acquisition. The clinician may collect the relevant data by interviewing and examining the patient, reviewing medical records, or interpreting the results of tests or studies. In the case of the test items used as the stimulus in this study, the relevant information is presented in the form of a brief clinical scenario. The clinician then creates a mental representation of the problem and generates hypotheses. If, for example, the question asked for “the most likely diagnosis” this mental representation might take the form of a differential diagnosis list. Finally, the clinician applies prior knowledge to the problem to either make a diagnosis by selecting the most likely choice from the hypothesis list or by identifying the information that is lacking to complete the diagnosis. The prior knowledge is sometimes referred to as an illness script—that is a succinct statement of the symptoms, objective data, and other characteristics associated with a specific diagnosis.

With this model for clinical reasoning in mind we might expect that test takers would begin by reading the item stem to gain an overview of the patient and related distinguishing features of the presentation. We would then expect the test taker to review the options and then return to the stem to review the specifics of the presentation to verify their hypothesis or reject it. Following this pattern in processing the information in the test item will not prove that the test taker is using an approach parallel to the real-world cognitive model we proposed, but using a substantially different process may prove that he or she is not following our proposed cognitive model. For example, if a test taker moves quickly to review the options and then frequently moves between the options and the stem it may suggest that the test taker is attempting to logic from the options to the answer rather than beginning by building a hypothesis based on the presented information. If this latter pattern proved to be a successful approach, it could certainly change our understanding of what the items measure, and it may undermine our confidence that the scores can be validly interpreted as representing proficiency in clinical reasoning.

Again, unlike previous studies that have used eye-tracking data to evaluate the cognitive models used by test takers in responding to examination items, we do not start with predetermined view of which features are most important. Instead, we allow machine learning algorithms to identify the features that are most useful for discriminating between test takers who respond correctly and those who respond incorrectly. We then compare the two problem-solving approaches to the general cognitive model presented in the previous paragraph. We focus on this distinction between correct and incorrect responses because the real-world cognitive model we have proposed relates to successful real-world problem solving. If a test taker lacks an illness script for the particular condition presented in the stem, it would not be surprising to see him or her adopt a less structured response process and ultimately respond incorrectly.

## Method

Implementation and evaluation of the machine-learning approach we used in this study was conducted in several steps. First, the test takers responded to the sample of items and the eye tracker recorded a range of gaze features. A model using all the recorded features was then trained to predict correct and incorrect responses and evaluated against several baseline models. An automatic feature selection procedure was then implemented to identify the most predictive features and evaluate whether these features improve the classification accuracy for correct and incorrect responses. The selected features were then analyzed to identify the problem-solving patterns they reveal. The paper concludes with a discussion of how these results contribute a comprehensive evaluation of the validity of score interpretations based on test items of this kind.

### Data

All participants completed a set of 40 MCQs sampled from the National Board of Medical Examiners’ (NBME) Clinical Mastery Series for Internal Medicine. This examination is built to the same content specifications as the NBME Clinical Science Subject Exams. The items were selected out of 50 items from a randomly selected self-assessment form, where lengthy items containing images or very long tables were excluded. This was done to present the items on the screen without the need for scrolling, which simplified interpretation of the results. These items all had either five or six answer options; The psychometric characteristics of the items were available from use in previous operational test administrations, where the mean p-value for the sample was 0.73 (SD = 0.12) and the mean point biserial correlation was 0.18 (SD = 0.08).

Data were recorded using an EyeLink® Portable Duo eye tracker with a sampling rate of 1000 Hz and a degree of visual angle < 0.5°; this is a faster recording rate than has been used in the previously described studies and allows for more reliable recording of quick eye movements which may not have been captured in previous studies. The recordings were performed in a video-based mode, where no equipment was attached to the participants. Data were extracted using EyeLink Data Viewer software package (SR Research Ltd., version 4.1.63) with its default setting for fixation duration threshold.

**Fig. 1:**
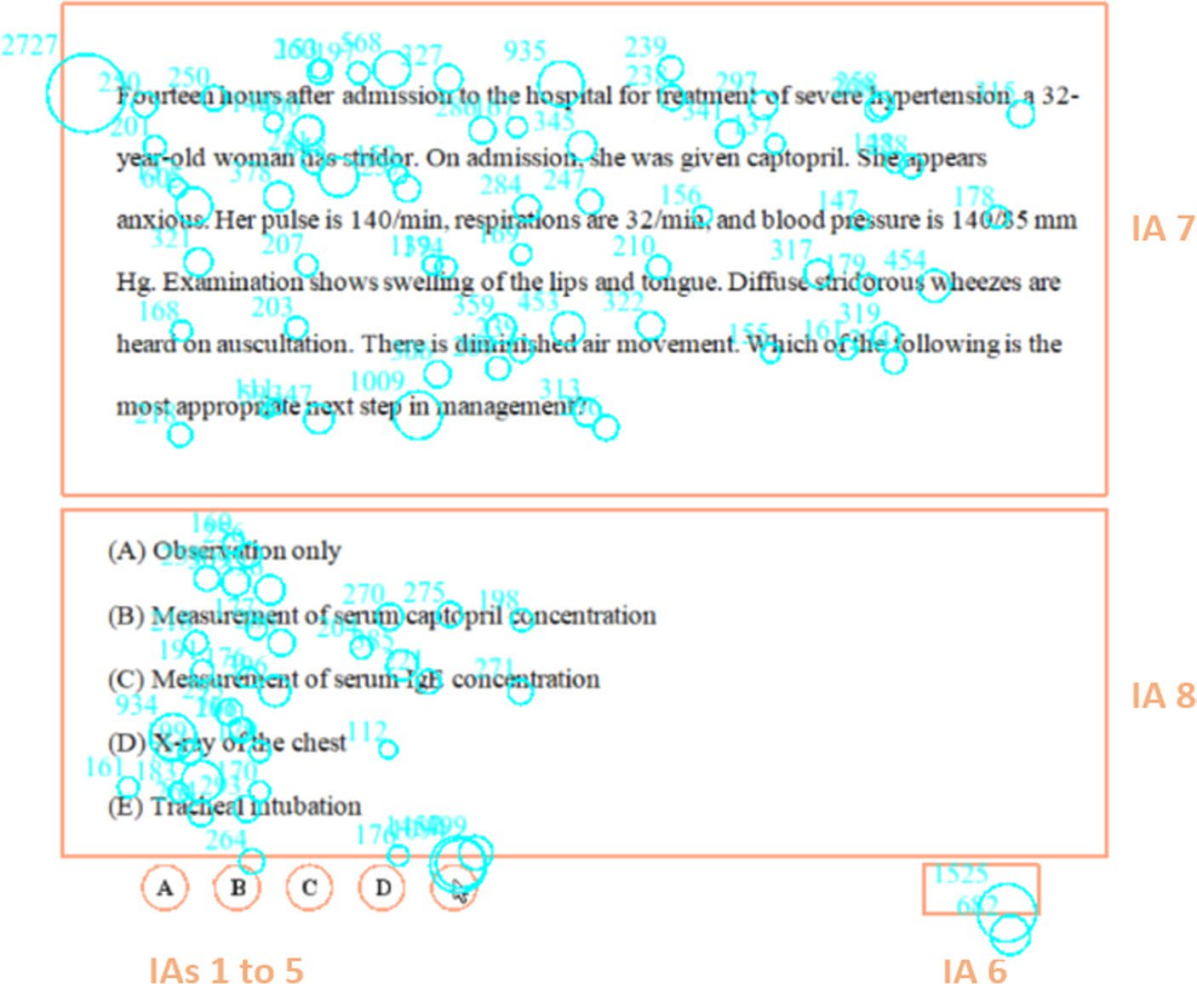
Example of gaze data from one participant over a practice item. The blue circles represent individual fixations, while the orange frames represent interest areas (IAs)

Two of the 40 items were designated as practice items used to familiarize the test takers with the format of the experiment. These practice items were not included in the analysis of the results. These items typically include information about a patient’s symptoms, history, a description of physical findings, and for some items the results of diagnostic studies. The task of the examinee was to select the most appropriate treatment, diagnosis, or course of action. Figure 1 shows a sample item overlaid with the gaze data from one individual. The gaze data and other annotations are explained in the following paragraphs.

The participant sample consisted of 26 students from US medical schools. The students were compensated for their time. The invitation to participate was available to all students from US medical schools who had passed USMLE Step 1 and the USMLE Step 2 Clinical Skills examination. All the students matriculated at medical school on (or before) August 2016, so at the time the data were collected (February and March 2020) they were in the final months of their fourth year of training. Additionally, participants had to be native English speakers to exclude potential confounding effects related to varying degrees of English proficiency and were screened for eye conditions that could interfere with interpretation of the study results. Because the experiment was conducted in Philadelphia, PA, our volunteers were all from local schools. Our protocol was reviewed and received IRB approval prior to data collection.

### Features

Once the data were collected, gaze-related features were extracted using the EyeLink Data Viewer software package (SR Research Ltd., version 4.1.63). These were extracted once for the stem region and once for the options region (noted in Fig. 1 as IA7 and IA8). For the benefit of readers who are not familiar with eye tracking technology, the following definitions will help with interpreting the feature descriptions that follow:

*Fixation* – a stable position of the eye gaze over an object of interest. Individual fixations are represented as blue circles in Figure 1 and their size corresponds to their duration measured in milliseconds. Fixation analysis usually focuses on the location and duration of fixations, as well as their sequential order. For example, when reading, complex parts of the text require longer fixations or a higher number of fixations.

*Saccade* – the movement the eyes make between individual fixations (i.e. fast jumps from one location to another).

*Interest Areas (IA)–* areas in the stimulus that are relevant to answering the research question. These are defined by the researcher and refer to the area of the stem and to the area of the answer options in the case of this study. Other areas that were present on the screen but excluded from the analysis were those of the buttons associated with selecting an answer option and the “Save” button which recorded the selection (Figure 1).

*Fixation Run* (also known as *Pass*) – a sequence of fixations from the start of entering an interest area until the area is left. For example, if the stem is visited three times, where after each time the eyes leave the stem to move to another region, then the stem area has a run count of three. Each run may consist of multiple fixations.

*Trial* – In the context of this study, a trial is defined as a test taker interacting with a single item.

*Interest Area ID* – the sequential number of an interest area (see Figure 1).

The descriptions of the different gaze features (as well as several non-gaze features) used in our experiments are presented in Table 1. It is important to note that these features were extracted for both the stem and the options regions, as well as, in certain cases, for specific fixations, saccades, and runs, as noted in the first column of Table 1 (e.g., once for the first fixation, once for the second fixation, etc.). This resulted in a total of 119 individual features (denoted as variables within Table 1).

**Table 1:** Features used in the study and their definitions

Feature group	Features	Feature definitions
*Non-gaze* *features*	Item ID	The unique ID of an item
	Participant ID	The unique ID of a participant
	Number of response options	The number of answer options for an item (either 5 or 6)
	Overall Response Time	The response time for an item measured in seconds from the moment the item was first displayed until the moment it was left (each item was displayed only once)
*Trial features*	Trial Dwell Time	The summed duration of all fixations within the trial (participant-item encounter)
	Trial fixation count	Total number of fixations in the trial
	Trial total visited IA count	Total number of unique interest areas (IAs) visited in the trial
*Generic gaze features* Stem and options 6 features × 2 regions = 12 variables	Dwell time	The summation of the duration across all fixations on the IA
	Dwell time %	Percentage of trial time spent on the current IA
	Fixation %	Percentage of all fixations in a trial falling in the current IA
	Fixation count	Total number of fixations falling in the IA
	Run count	Number of times the IA was entered and left (runs)
	Skip	An IA is considered skipped if no fixation occurred in first-pass reading
*Fixation* *Features* 1^st^, 2^nd^, 3^rd^, and Last fixations Stems and options 3 features × 4 fixations × 2 regions = 24 variables	Fixation duration	Duration of the first/second/third/or last fixation event that was within the current IA
	Fixation run	The index of the run that the particular fixation belongs to (first/second/third/or last fixation)
	Fixation time	Start time (in milliseconds relative to the start of the current trial) of the first/second/third/or last fixation to enter the current IA
*Saccade features* 1^st^ and Last saccades Stems and options 5 features × 2 saccades × 2 regions = 20 variables	Saccade amplitude	Amplitude (in degree of visual angle) of the first /last saccade entering into the current IA
	Saccade angle	Angle between the horizontal plane and the direction of the first/last saccade entering into the current IA
	Saccade Index	Ordinal index of the saccade in a trial which first/last landed within the current IA
	Saccade start time	Start time of the saccade that first/last landed within the current IA
	Saccade end time	End time of the first/last saccade that landed within the current IA
*Run features* 1^st^, 2^nd^, 3^rd^, and Last runs Stems and options 5 features × 4 runs × 2 regions = 40 variables	Run start time	Start time of the first/second/third/last run of fixations in the current IA
	Run end time	End time of the first/second/third/last run of fixations in the current IA
	Run dwell time	Dwell time of the run (i.e., the sum of the duration of all fixations in the first/second/third/or last run of fixations within the current IA)
	Run fixation %	Percentage of all fixations in a trial falling in the first/second/third/ last run of the current IA
	Run fixation count	Number of all fixations in a trial falling in the first/second/third/last run of the current IA
*Regression features* Stems and options 8 features × 2 regions = 16 variables	Regression in	Whether the current IA received at least one regression from later IA (usually entering the stem from the options region)
	Regression in count	Number of times an IA was entered from an IA with a higher ID
	Regression out	Whether regression(s) was made from the current IA to earlier IAs prior to leaving that IA in a forward direction
	Regression out count	Number of times an IA was exited to an area with a lower ID number before a higher ID area was fixated in the trial
	Regression out full	Whether regression(s) was made from the current IA to earlier IAs (binary feature). Note that *Regression Out* only considers first-pass regressions whereas *Regression Out Full* considers all regressions, regardless whether later IAs have been visited or not
	Regression out full count	Number of times an IA was exited to an area with a lower ID number
	Regression path duration	The summed fixation duration from when the current IA is first fixated until the eyes enter an IA with a higher ID number
	Selective regression path duration	Duration of fixations and refixations of the current IA before the eyes enter an IA with a higher ID number

As noted in the first column “feature group”, the gaze features were extracted once for the stem and once for the options regions, as well as, in certain cases, for specific fixations, saccades, and runs. For example, we extract “fixation duration” for the first fixation within the stem region, the second fixation within the stem region, the third fixation within the stem region, and the last fixation within the stem region; then we do the same for the first, second, third, and last fixations in the options region. This is denoted as “1st, 2nd, 3rd, and Last fixations; Stems and options” in the column “feature group”

### Analysis

The main study analysis consists of three parts: (1) training a machine-learning classifier using the 119 features from Table 1 to predict whether a given response is correct or incorrect, (2) performing feature selection to identify the combination of features that provides the best predicted classification, and (3) examining the patterns associated with correct and incorrect responses based on the combination of selected features. These steps are described in more detail in the sections that follow.

#### Classification

From the total of 988 trials (test taker-item encounters), a random 20% were separated as a test set to be used for evaluation and the remaining 80% were used to train a machine-learning-based classifier. Since the main objective of this study is to identify predictors that can reliably distinguish between patterns corresponding to correct and incorrect responses, our primary metric of interest is classification precision (also known as specificity), ranging between 0 and 1. For example, a precision score of 1 for the class of correct responses indicates that 100% of the responses that were predicted correct were indeed correct. We also report recall (sensitivity), also ranging between 0 and 1, where a recall of 1 for the class of correct responses indicates that of the set of responses that were indeed correct, all were predicted correct. Finally, we report a metric representing the harmonic mean of precision and recall, known as F1 score (ranging between 0 and 1, with 1 indicating perfect classification) (Davis & Goadrich, 2006). Precision and Recall are computed as follows, where TP stands for true positives, FP stands for false positives, and FN stands for false negatives:

$$Precision = \frac{TP }{TP + FP}$$

$$Recall = \frac{TP}{TP + FN}$$

Once the training and test sets were separated, several common classifiers were fit to the training data using the *Scikit-learn* library in *Python 3.6* (e.g., random forests, support vector machines*,* and a gradient boosting classifier, among others). For brevity, we only report results from the best-performing classifier, logistic regression. This is also the classifier most likely to be familiar to readers. Before evaluating the full model using the features from Table 1, several baselines were defined to benchmark the classification performance of the gaze features:

- *Majority class baseline*: Owing to class imbalance (732 correct responses and 256 incorrect ones), assigning an example to the majority class is more likely to turn out to be correct. The majority class baseline simply assigns all test-set examples to the correct response category and measures the resulting Precision, Recall, and F1 scores. This is sometimes refered to as the *best *a priori classification.
- *Response time baseline*: Previous research suggests that test takers typically spend relatively more time on items they answer incorrectly (e.g., Harik et al., 2020). This baseline tests the extent to which response time can explain the differences between correct and incorrect responses.
- *Number of options baseline:* The items in our sample had either five or six possible answer options. This baseline tests the extent to which the number of options in an item can predict correct and incorrect responses.
- *Participant baseline:* Since our participant sample does not include specific groups of low-proficiency and high-proficiency test takers, this baseline tests whether knowing what participant responded in a specific trail is useful in predicting its category correctly.
- *Combined baseline:* All of the previous baselines consist of a single feature used as a predictor in a logistic regression model. The combined baseline includes all these features as predictors in a single model. As the gaze features are added later, the combined baseline allows assessing what part of the full model performance can be attributed to the addition of the gaze features and the interactions between features.

#### Feature selection

To select the most predictive features, a Least Absolute Shrinkage and Selection Operator (LASSO) feature selection was applied to the training set. LASSO is an embedded method for feature selection which performs regularization through penalizing the coefficients of the regression variables (Tibshirani, 1996). Some of the variable coefficients are shrunk to zero, while those that still have a non-zero coefficient after the shrinking process are selected to be part of the model. This procedure was applied to the training set using the *LassoCV* command from *Scikit-learn* in *Python 3.6*, with the number of cross validation folds set to 20. The method tests different levels of penalization and selects the best performing one using the data from some of the folds as the training set and the data from the remaining folds as a test set; in essence, it uses a cross validation procedure within the training set to produce robust results.

## Results

In the sections that follow we will present results on classification accuracy, feature selection, and analysis of the selected features.

### Classification

Table 2 presents the performance of the baseline models and the full model based on all features with respect to precision, recall, and weighted F1.^3^ As the table demonstrates, all of the baseline models using a single variable regress to a majority class assignment; that is, the algorithm learns that assigning all instances to the majority class leads to a better performance than using the individual predictor (Precision = 0.56, Recall = 0.75). The combined baseline model outperforms the majority class model, improving precision from 0.56 to 0.64, while maintaining recall at a similar level. Adding the gaze features results in a model that performs noticeably better with precision of 0.75 and recall of 0.77. As was mentioned in the method section, these results are based on a cross validation sample, so the improved prediction is not explained by the increased number of predictors in the model.

**Table 2:** Results for the performance of the different baselines and the full model

Model	Precision	Recall	Weighted F1
Majority class	.56	.75	.64
Response time	.56	.75	.64
Number of Options	.56	.75	.64
Participant ID	.56	.75	.64
Combined baseline	.64	.74	.65
Full model	.75	.77	.74

The performance of the gaze features provides a clear evidence that they contain signal relevant to the classification of correct and incorrect responses. While this result shows that there are different eye-movement patterns (response processes) associated with correct and incorrect responses, it does not provide specific insights into these patterns. Identifying these patterns is the purpose of the feature selection procedure.

#### Feature selection

**Fig. 2:**
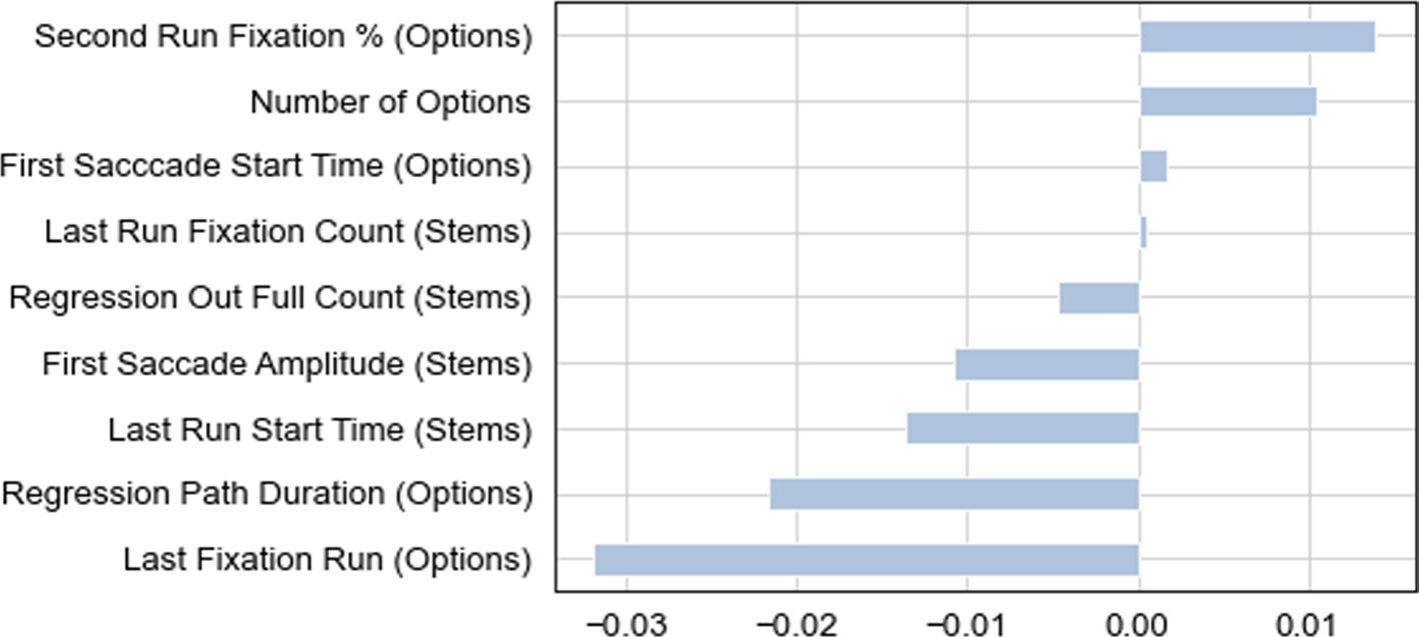
Selected features and their indices. The positive coefficients indicate variables that are associated with an increased probability of a correct response and the variables with negative coefficients are associated with an increased probability of an incorrect response. The larger the coefficient, the stronger the relationship between the variable and the outcome

The LASSO feature selection resulted in the selection of nine variables presented in Fig. 2. The positive coefficients indicate variables that are associated with an increased probability of a correct response and the variables with negative coefficients are associated with an increased probability of an incorrect response. The larger the coefficient, the stronger the relationship between the variable and the outcome.

**Fig. 3:**
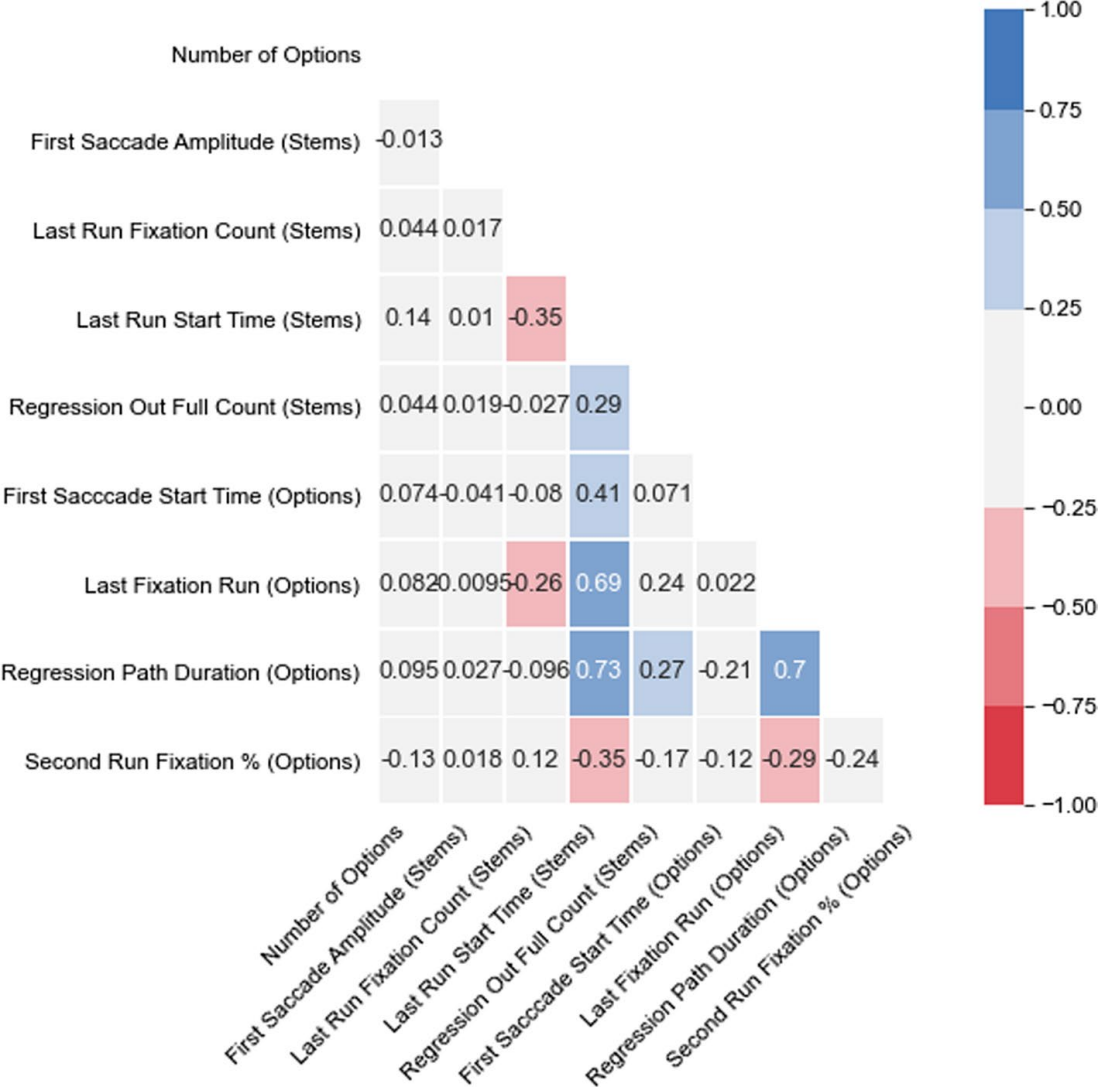
Correlations between the selected features. Most of the selected features are not highly correlated with each other, indicating that the individual features carry substantially independent information

When interpreting the significance of the selected features, it is important to note that they were selected based the signal they contributed when interacting with each other, rather than their merit as individual predictors. Nevertheless, as can be seen from the correlation matrix presented in Fig. 3, the majority of the selected features are not highly correlated, indicating that the individual features carry substantially independent information. When moderate correlations do exist (0.41, 0.69, 0.73) it is for features that are are likely to be linked to the total amount of time a test taker spent on an item.

**Fig. 4:**
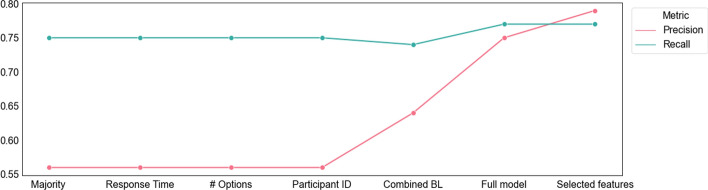
Precision and Recall for the different models. Higher values indicate better classification accuracy

To evaluate the quality of the feature selection step, Fig. 4 compares a model using only the selected features to the full model and the five baselines. Again, these results are cross validated. The feature selection was performed on the training set; the evaluation was performed on the test set. This resulted in the highest precision of 0.79 and recall of 0.77.

Analysis of the nine selected features (shown in Fig. 2) reveals that the most important differences between correct and incorrect responses were in the processing of the options region. These results are summarized in Table 3.

**Table 3:** Selected features per region

Importance	Correct responses—stems	Correct responses—options	Importance
Low	Slightly higher number of fixations belonging to the last run on the stem	Higher percentage of fixations belonging to the 2^nd^ run over the options compared to other runs Later start time of the first saccade in the options area, indicative of starting to read the options later	Medium
	Incorrect responses—stems	Incorrect responses—options	
Medium	Later start of the last run over the stem Slightly more cases where the eyes moved vertically or diagonally after the first fixation on the stem Slightly more cases where the eyes move from the stem to the option selection buttons	More runs over the options More time spent on the options region	High

#### Processing of the options region

**Fig. 5:**
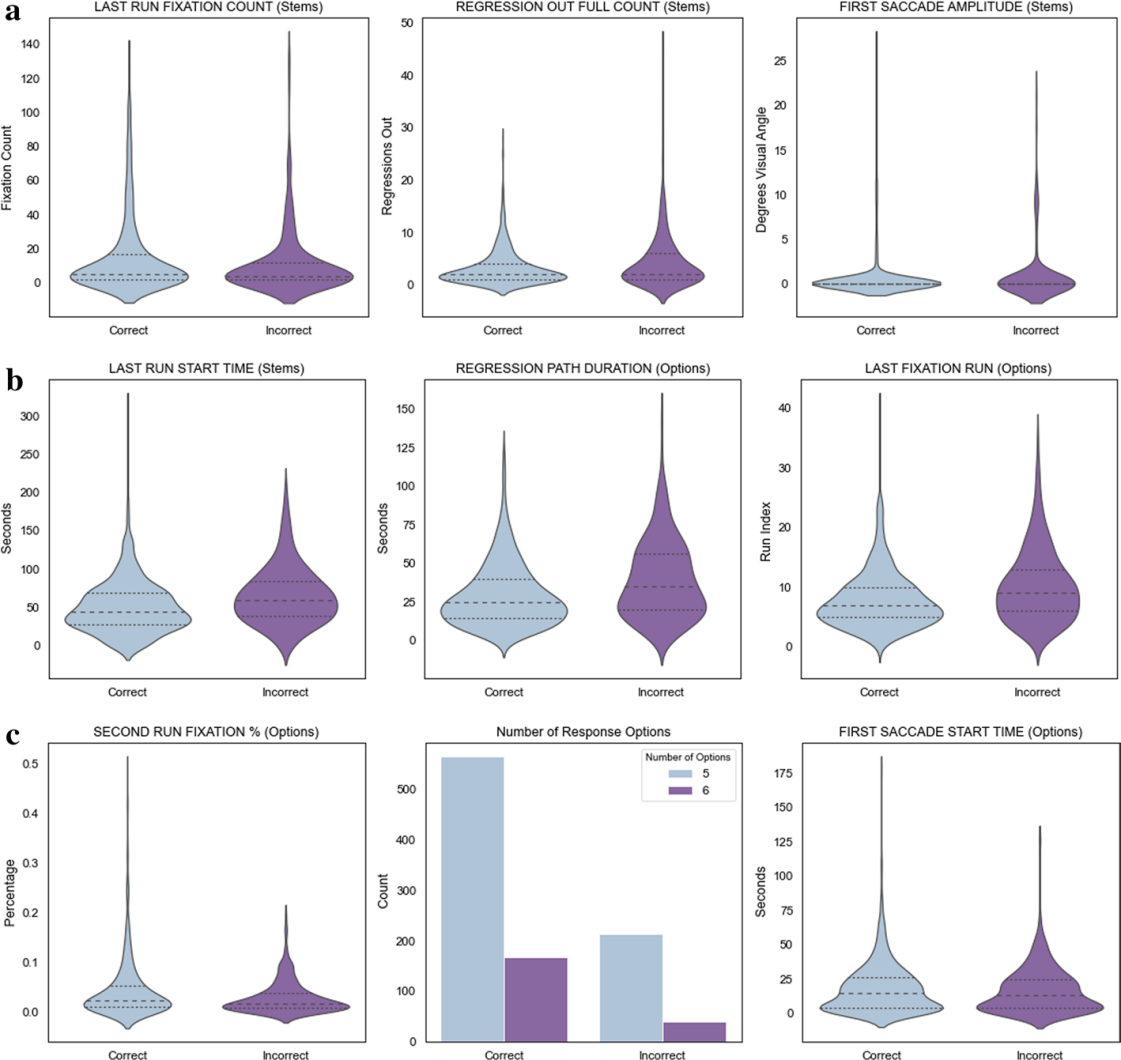
a Distribution of the selected features across correct and incorrect responses (1—3). Wider sections of the violin plot represent a higher probability that the features will take on the given value; dotted lines represent quartiles. b: Distribution of the selected features across correct and incorrect responses (4 – 6). Wider sections of the violin plot represent a higher probability that the features will take on the given value; dotted lines represent quartiles. c: Distribution of the selected features across correct and incorrect responses (7 – 9). Wider sections of the violin plot represent a higher probability that the features will take on the given value; dotted lines represent quartiles

The distributions of the nine selected features over the categories of correct and incorrect responses are presented in Fig. 5a, b, c. The two features with the strongest predictive power are *Last Fixation Run—Options* and *Regression Path Duration—Options*, shown in Figs. 6a and 6b. The *Last Fixation Run—Options* feature indicates that incorrect responses are characterized by test takers returning to the region containing the options more frequently than is the case for correct responses.

The *Regression Path Duration—Options* feature indicates that test takers who answer incorrectly tend to spend more processing time after their gaze first enters the options region: that is the time from when they first look at the options until they respond and exit the item is longer for test takers who answer incorrectly. Combined with information about other variables presented below, this feature may suggest that the options region is accessed earlier in cases that resulted in incorrect responses in addition to incorrect responses having longer overall response times.

In contrast to incorrect responses, which tend to have more passes over the options region, as shown in Fig. 5c, correct responses were associated with a higher number of fixations during the second pass over the options (*Second Run Fixation % —Options*). In addition, the first time the eyes moved inside that region happened later for correct reponses than for incorrect ones (*First Saccade Start Time—Options*).

The only non-gaze variable that was significantly related to classification was the number of options in the item. For the sample of items used in this study five-option items were slightly easier than six-option items. This would seem to be an artifact of the specific items selected for the study.

Overall, the data related to the options region suggest that correct responders accessed that region later but concentrated on the options earlier. Incorrect responders on the other hand, tended to access the options earlier but needed more passes over that region, indicating that they were moving back and forth between the stem and the options more frequently.

### Processing of the Stem Region

The features related to the stem region had relatively lower importance for classifiction but corroborrated the idea that the eyes accessed the options region earlier in cases of incorrect responses. For example, *First Saccade Amplitude—Stems* shows that incorrect answers are associated with a larger number of first saccades in the stem that have a large visual angle. That is, incorrect responses tend to be associated with instances in which the eyes likely moved vertically or diagonally as opposed to horizontally on the line after the first fixation on the stem. This would be consistent with a less structured approach to reading the item: browsing different areas of the stem or moving back and forth between the stem and options as opposed to processing the information sequentially.

In line with the evidence that incorrect responses had more passes and overall longer response times, *Last Run Start Time—Stems* shows that incorrect responses are associated with a final pass of the eyes over the stem region happening relatively later than with correct responses. The correct responses on the other hand, had a slightly higher number of fixations belonging to the last run. This information paints a picture of higher response times for the incorrect responses due not only to longer fixations in given regions but due to *more passes* over the regions. This may imply that test takers who are uncertain about the answer are scanning for clues rather than following a hypothesis-driven search procedure.

Finally, the information revealed by the *Regression Out Full Count—Stems* feature corresponds to the number of times an interest area was exited to an area with a lower ID number. In this study, the only areas with lower ID numbers than the stem were the buttons at the bottom of the screen corresponding to the selection of the different answer options, as well as the “Save” button which is pressed to record the selected option and move on to the next item (Fig. 1). Therefore, this feature reveals that incorrect responses are associated with more cases in which the eyes move directly from the stem to an answer selection button.

## Discussion

The analysis of the selected features reveals that incorrect responses are characterized by more passes of the eyes between the stem and the options. Since response time alone was not a highly predictive variable and since the addition of the gaze features to response time and other baselines boosts performance, it can be concluded that the pattern of incorrect responses is not one where the test-taker simply spends longer looking at the item; rather, it is a pattern where the test-taker accesses the options region earlier and makes multiple passes across the stem and the options. This behavior suggests that incorrect responses may be associated with working from the options to the stem instead of first reading the stem carefully and then processing the options after having formed a (preliminary) hypothesis. This pattern is also consistent with the idea that test takers who respond incorrectly simply use a more random approach to processing the material in the item; they may scan the item numerous times hoping to find a clue or recognize a pattern that was not initially apparent.

By comparison to this more random approach, test takers who answered correctly appear to use a cognitive model that is generally consistent with the real-world model described previously (see Bowen, 2006). They tend to review the material in the stem more carefully, review the options later, and then make fewer moves between the options and the stem. This pattern would be consistent with a model in which they systematically evaluate the information about the patient, formulate a hypothesis, review the options to identify the correct answer based on their hypothesis, and then return to the stem to verify their initial impressions and check that they have not missed evidence that contradicts their hypothesis. The fact that our results are generally consistent with the proposed real-world model certainly does not prove that test takers who respond correctly are using the same cognitive model that successful clinicians use in practice (if that were possible, the task of constructing a validity argument would be relatively trivial). Again, evidence of the type we have collected is much more likely to provide a definitive rejection of the intended interpretation of test scores than definitive support. For example, if we had observed that successful test takers immediately accessed the options and then moved frequently between the options and the stem, the validity of the resulting score interpretations would be significantly undermined.

It is important to note that the findings of this data-driven study complement the findings of prior eye-tracking work presented in our literature review and are in line with the results from studies with other types of process data. For example, fMRI (functional Magnetic Resonance Imaging) data have shown functional differences between correct vs. incorrect answers and guessing vs. not guessing for 17 physicians answering internal medicine MCQs (Durning, et al., 2012). We note that these types of inferences are substantially different from inferences that can be made using other types of process data such as timing information (Margolis & Feinberg, 2020), which has been used to identify disengaged test-takers (Wise, 2015, 2017), item exposure (Lee & Wollack, 2020), or that the imposed time limit is impacting the measurement of test taker proficiency (Harik et al., 2020).

Although the present results are consistent with expectations, it is important to consider the extent to which these results are likely to be generalizable. The simple answer is that we cannot be sure until follow-up studies are completed with larger test-taker samples in actual testing settings. In an operational testing setting test takers may be more motivated to perform well and may alter their test-taking strategies accordingly. The participants in this study were compensated for their time and were provided with detailed feedback on their performance. They also completed the test in the presence of one of the experimenters. The presence of the experimenter and their interest in feedback may have been motivating factors. We clearly have no evidence about whether the participants performed up to their potential. We do know that the mean performance for the participants was modestly higher than the performance on the same items when they were used in operational testing (mean p-value of 0.73 for operational testing and 0.74 for the participants in this study). We also know from the timing data recorded for each item that none of the participants responded so quickly that we should question whether they were seriously engaged with the test material.

It is tempting to think that the results of this paper might provide insight into building optimal testing strategies for medical students. At this time, the results do not support designing such strategies. The present results describe some of the behaviors that are associated with responding correctly to the types of clinical reasoning items included in this study. The results do not demonstrate a causal relationship. It may be that the behaviors we describe as being associated with responding correctly occur because the test takers have the prior knowledge to reason from the patient information to the correct answer. If this is true, applying this more structured approach to responding would not be helpful to individuals lacking the specific knowledge. The results do suggest that in general a strategy that focuses on examining the options before evaluating the patient information is not, in general, a successful strategy. This is consistent with results reported by Yaneva et al. (2021), showing that test takers that used that approach performed relatively poorly on clinical reasoning items. That said, the critical issue in a testing strategy is that it improves the test takers probability of success conditional on both the test takers general level of proficiency and his or her knowledge of the specific content area tested by the item. Differences in the probability of success associated with alternative response processes may provide a misleading estimate of the changes in these more specific conditional probabilities.

In interpreting these results there are a number of points that should be kept in mind. The first is that we have described the contrasting processes used by successful and unsuccessful test takers, but these different processes are associated with a change in the likelihood of responding correctly—the process differences are not deterministic, they are probabilistic. The second consideration is that while some previous studies have reported on differences in processing for more and less proficient test takers, our study focuses on correct and incorrect responses. As such, our results suggest that the same individual my use different approaches for different items. The fact that the process data substantially improved prediction above and beyond the baseline model, which included the test taker as a variable, strongly suggests that test takers do change the process they use across items. It appears that when individuals have the required prior knowledge (e.g., a relevant illness script), that knowledge allows them to recognize the pattern of patient characteristics presented in the stem, and they are able to use that knowledge to form a hypothesis (e.g., a differential diagnosis list) and evaluate that hypothesis against reasonable alternatives. Again, this would be consistent with the real-world model we presented. When faced with a different case, that same test taker may be unable to apply a systematic and efficient problem-solving approach, and so adopt an alternative, seemingly more random strategy. In some ways this is consistent with the behavior of clinicians in practice, in which an otherwise competent clinician occasionally misses a diagnosis because of gaps in knowledge. As we noted, the argument here is not that real-world responding is the same as responding to the test item, but simply that some of the critical cognitive skills required for the real-world response are required to successfully respond to the test item.

The results from this study are consistent with some of the findings from previous studies, such as the evidence that unsuccessful problem-solvers tend to experience difficulties in decoding the problem and recognizing the relevant factors (Tsai et al*.,* 2011) and that high-performing test takers have fewer fixations across tasks (Hu et al., 2017). The present study also goes beyond previous work by using machine-learning procedures to evaluate all the features recorded by the eye-tracking system and identify those that distinguish successful from unsuccessful responses. This eliminates the kind of confirmation bias that can exist when a researcher selects a small number of features for evaluation. Again, the present study also differs from most previous research in that we do not assume that the response process is a characteristic of the individual; we allow for the possibility that the process may change as a test taker moves from one item to another.

- The authors were employees of the National Board of Medical Examiners at the time the research was conducted. There were no additional sources of funding for this work.
- The authors have no conflicts of interest to declare that are relevant to the content of this article.
- This study was performed in line with the principles of the Declaration of Helsinki. The study was granted exemption status by the Institutional Review Board of the American Institutes for Research (Date: 10/15/2019 /No. EX00496)

## Appendix A

See Table 4.

**Table 4:** Summary of previous work on the use of eye tracking in assessment

Study	Subjects	Items	Number of eye-tracking features	Sampling rate
Hegarty et al. (1992)	32	18 math items × 4 conditions (1 condition per subject)	No specific features; Distinction between the amount of time spent reading the item for the first time versus re-reading it	60 Hz
Tai et al. (2006)	6	18 items from biology, chemistry and physics	4 features (fixation locations, fixation durations, saccades, and saccadic durations)	60 Hz
Tsai et al., (2011)	6	One visually based science problem with 4 images	1 feature (fixation duration) + heatmaps + analysis of the sequences in which different item components were visited	60 Hz
Hu et al. (2017)	28	2 PISA test problems involving drag-and-drop: A rule-induction problem requiring the arrangement of pictures and an interactive simulation problem requiring an efficient way to solve a task	4 features (fixation duration, fixation count, visit duration, and visit count)	120 Hz
Maddox et al. (2018)	14	Standard format of the Programme for the International Assessment of Adult Competencies (PIAAC) (number of items not explicitly reported)	Descriptive scanpath analysis was performed to determine the order of the regions the respondent visited, and for how long. Pupil data were used to validate examinee engagement	60 Hz
Langenfeld et al. (2020)	18	32 multiple choice scored items based on 14 unique graphics + six pretest items based on three graphics	Sequence analysis of fixations and their durations (number of fixations, total fixation time, and time to first fixation) + Heatmaps + Pupillometry data	60 Hz

## Footnotes

1 Gaze features are eye-tracking variables related to fixating the eyes on a given location (“gaze fixations”) or moving the eyes from one location to another (“saccades”).

2 For comparison, the sampling rate of the device used in this study is 1000 Hz, leading to more fine-grained eye movement recordings.

3 Weighted for class imbalance.
